# From Childhood Icterus to Adolescent Gallstones: Clinically Diagnosed Crigler‐Najjar Syndrome Type II

**DOI:** 10.1002/ccr3.72080

**Published:** 2026-02-18

**Authors:** Danish Kumar Goswami, Barkha Goswami, Samiullah Shaikh, Abdul Ghani Rahimoon, Rakesh Soni, Kamil Ahmad Kamil

**Affiliations:** ^1^ Department of Internal Medicine Liaquat University of Medical and Health Sciences Jamshoro Sindh Pakistan; ^2^ Mirwais Regional Hospital Kandahar Afghanistan

**Keywords:** cholelithiasis, Crigler–Najjar syndrome, gastroenterology/hepatology, general medicine, genetics, jaundice, unconjugated hyperbilirubinemia

## Abstract

Crigler–Najjar syndrome type II (CNS‐II) is an uncommon cause of persistent unconjugated hyperbilirubinemia resulting from partial deficiency of hepatic UDP‐glucuronosyltransferase activity. We report the case of a 19‐year‐old male who presented with intermittent jaundice since childhood and recent worsening of scleral icterus. Laboratory evaluation revealed isolated unconjugated hyperbilirubinemia with normal liver enzymes. Genetic testing was unavailable; however, serum bilirubin levels declined significantly following phenobarbital therapy, confirming the diagnosis of CNS‐II. Abdominal ultrasonography demonstrated gallstones, indicating chronic bilirubin supersaturation secondary to longstanding hyperbilirubinemia. The patient was managed conservatively with phenobarbital and counseling on avoiding precipitating factors such as fasting and hepatotoxic drugs. This case underscores the importance of recognizing CNS‐II as a differential diagnosis in young adults with isolated unconjugated hyperbilirubinemia and cholelithiasis. It also highlights phenobarbital responsiveness as a valuable diagnostic tool in settings lacking molecular testing.

## Key Clinical Message

This case highlights an uncommon adult presentation of Crigler–Najjar syndrome type II, complicated by cholelithiasis, underscoring the need to consider inherited disorders of bilirubin metabolism in persistent unconjugated hyperbilirubinemia. Recognizing phenobarbital responsiveness remains crucial for diagnosis in resource‐limited settings where genetic testing is not readily available.

## 1 Introduction

Crigler–Najjar syndrome (CNS) is a rare autosomal recessive disorder of bilirubin metabolism caused by mutations in the uridine diphosphate‐glucuronosyltransferase (*UGT1A1*) gene, leading to reduced or absent activity of the hepatic enzyme UGT1A1, thus resulting in unconjugated hyperbilirubinemia of varying severity. CNS is classified into two types: type I, which is severe and presents in the neonatal period with complete enzyme deficiency, and type II, which is milder and presents in late childhood or adolescence, characterized by partial enzyme activity and phenobarbital responsiveness [1, 2].

Patients with CNS‐II may develop complications such as cholelithiasis due to chronic bilirubin supersaturation and calcium–bilirubinate stone formation [3]. The global prevalence is estimated at approximately 0.6–1 per million live births [1], though the incidence is higher in regions with prevalent consanguineous marriages, such as South Asia [4]. Phenobarbital, a cytochrome P450 inducer, enhances the remaining activity of the UGT1A1 enzyme and has been shown to improve the clinical manifestations of the disease [5]. In many low‐resource settings, UGT1A1 gene testing is not readily available, and diagnosis relies on biochemical profiling and the documented reduction in unconjugated bilirubin after phenobarbital therapy [6].

We report a rare case of a young adult male with CNS‐II, complicated by gallstone formation, diagnosed clinically based on persistent unconjugated hyperbilirubinemia and a marked phenobarbital response. This case underscores the diagnostic value of a supervised phenobarbital trial and highlights the need to recognize CNS‐II in adolescents with long‐standing jaundice. This case is described in accordance with CARE guidelines [7].

## 2 Case Presentation

### 2.1 Presenting Complaints and Disease History

A 19‐year‐old male student with no history of tobacco use, alcohol consumption, or recreational drug use presented to the emergency department with right upper quadrant abdominal pain and persistent yellow discoloration of the eyes. On further inquiry, he reported a history of jaundice and cholelithiasis since the age of five. He noted that the icterus tended to worsen during periods of stress and intercurrent illnesses.

Despite multiple hospital admissions over the years and various conventional and alternative treatments, no definitive diagnosis had been established. His birth history was unremarkable, with no history of neonatal jaundice or need for phototherapy or exchange transfusions, and all developmental milestones were achieved on time. There was a positive family history of consanguinity in his parents, and one paternal cousin had also reportedly suffered from jaundice since birth. Based on these findings, the patient was admitted to the medical ward for detailed evaluation and further investigations.

**FIGURE 1:**
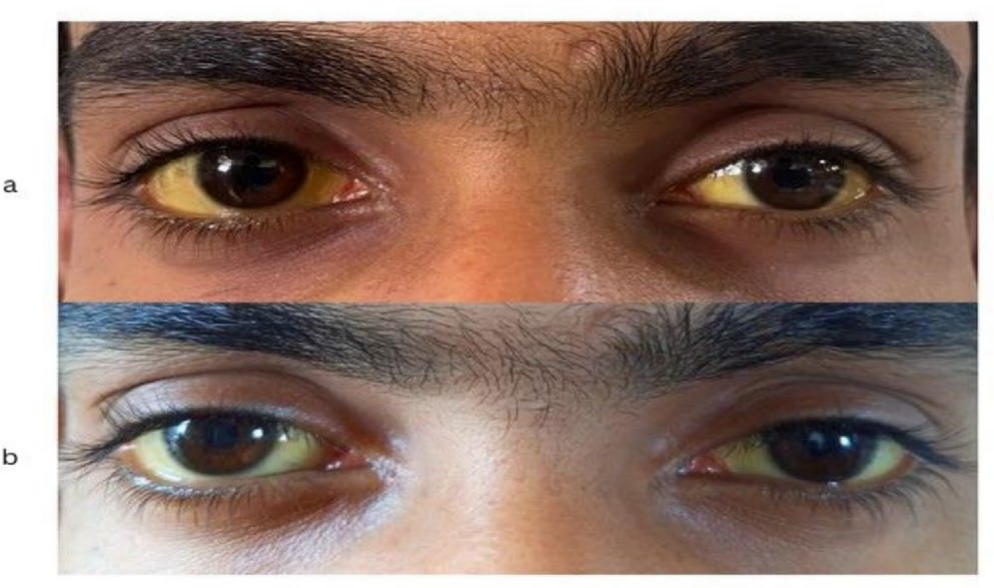
Clinical manifestation of a patient showing scleral icterus. (a) Marked scleral icterus in a 19‐year‐old male with long‐standing unconjugated hyperbilirubinemia before phenobarbital therapy. (b) Same patient four weeks after phenobarbital therapy, showing visibly reduced scleral icterus.

On physical examination, the patient had marked scleral icterus without peripheral stigmata of chronic liver disease (Figure 1a). The abdomen was soft and nontender, with no organomegaly, and the remainder of the systemic examination was unremarkable.

### 2.2 Lab Investigation and Imaging

Initial laboratory investigations revealed a total serum bilirubin of 23.51 mg/dL, with an indirect (unconjugated) fraction of 22.86 mg/dL and a direct (conjugated) fraction of 0.65 mg/dL (Table 1). Other liver function tests were within normal limits, and there was no evidence of coagulopathy. Complete blood count, renal function tests, and serum electrolytes were all within normal ranges. Viral serology for hepatitis B and C, as well as HIV, was negative. A comprehensive hemolysis workup, including direct Coombs test, lactate dehydrogenase (LDH), serum haptoglobin, and glucose‐6‐phosphate dehydrogenase (G6PD) levels, was unremarkable (Table 1). Hemoglobin electrophoresis showed normal hemoglobin patterns with normocytic, normochromic red blood cells. Abdominal ultrasonography demonstrated cholelithiasis within the gallbladder lumen without biliary ductal dilatation or obstruction (Figure 3).

**TABLE 1:** Laboratory results of the patient at presentation and after 4 weeks of phenobarbital therapy.

Parameter	Result at presentation (baseline)	Follow‐up after phenobarbital (4 weeks)
Hematology		
Hemoglobin	14.2	14.3
Total leukocyte count	7.4	7.1
Platelet count	265	252
Liver function tests (LFTs)		
Total bilirubin (mg/dL)	23.51	6.2
Indirect (unconjugated) bilirubin (mg/dL)	22.86	5.59 (≈70% reduction from baseline)
Direct (conjugated) bilirubin (mg/dL)	0.65	0.61
AST (U/L)	26	25
ALT (U/L)	29	33
ALP (U/L)	90	78
INR/prothrombin time	0.9	1.0
Renal function and electrolytes		
Serum creatinine	0.6	—
Blood urea nitrogen	18	—
Serum electrolytes (Na^+^, K^+^, Cl^−^)	Within normal limits	—
Viral serology		
HBsAg	Negative	—
Anti‐HCV	Negative	—
HIV	Negative	—
Hemolysis workup		
Reticulocyte count	1.0	—
Direct Coombs test	Negative	—
LDH	90	—
Serum haptoglobin	120	—
G6PD level	Within normal limits	—
Hemoglobin electrophoresis	Normal pattern; no hemoglobinopathy	—

Abbreviations: AST: aspartate aminotransferase; ALT: alanine aminotransferase; ALP: alkaline phosphatase; INR: international normalized ratio; LDH: lactate dehydrogenase; G6PD: glucose‐6‐phosphate dehydrogenase; HBsAg: hepatitis B surface antigen; Anti‐HCV: antibody to hepatitis C virus; HIV: human immunodeficiency virus.

**FIGURE 2:**
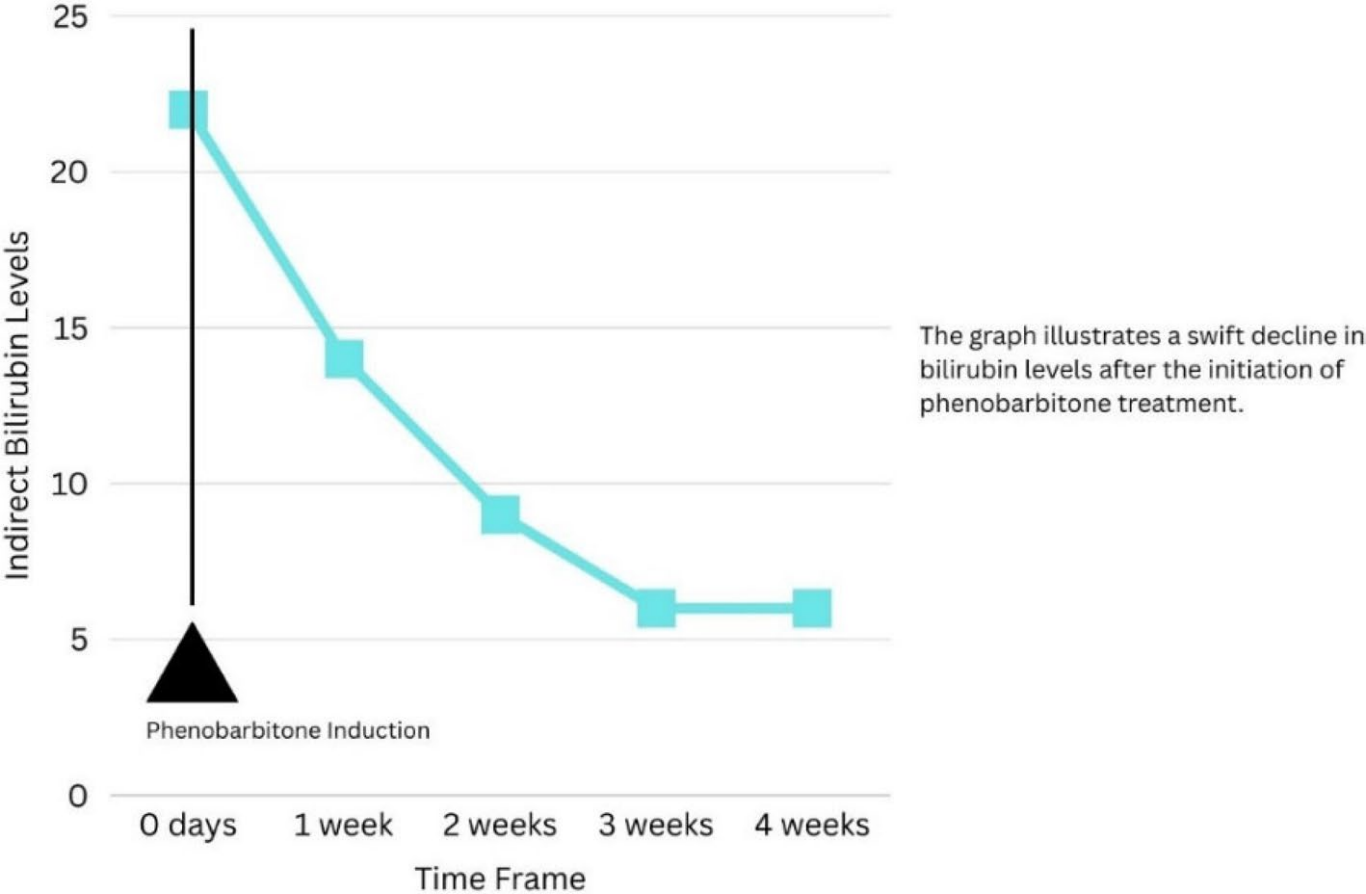
Serial trend in serum bilirubin demonstrating a marked decline in unconjugated (indirect) bilirubin following initiation of phenobarbital therapy, with approximately 50% reduction at 1 week and nearly 70% reduction at 4 weeks compared with baseline.

In view of isolated unconjugated hyperbilirubinemia with normal liver enzymes and no evidence of hemolysis, the main differentials considered were Gilbert syndrome and Crigler–Najjar syndrome type II (CNS‐II). Gilbert syndrome was considered less likely for two reasons. First, the patient's markedly elevated indirect bilirubin (22.86 mg/dL), which exceeds the typical levels (< 4.93 mg/dL) seen in Gilbert syndrome [8]. Second, the patient's long‐standing history of jaundice from early childhood, with multiple hospital visits, was more suggestive of CNS‐II than of Gilbert syndrome, which manifests as mild, intermittent episodes of scleral icterus or is discovered incidentally on blood tests, and usually does not lead to significant morbidity or repeated hospitalizations [9]. In our resource‐limited healthcare setting, genetic testing for *UGT1A1* mutations was not available and the patient's family declined private genetic testing due to considerable financial burden. The diagnosis of CNS‐II was therefore made on clinical grounds, biochemical profile, and the documented response to phenobarbital (Figure 2).

**FIGURE 3:**
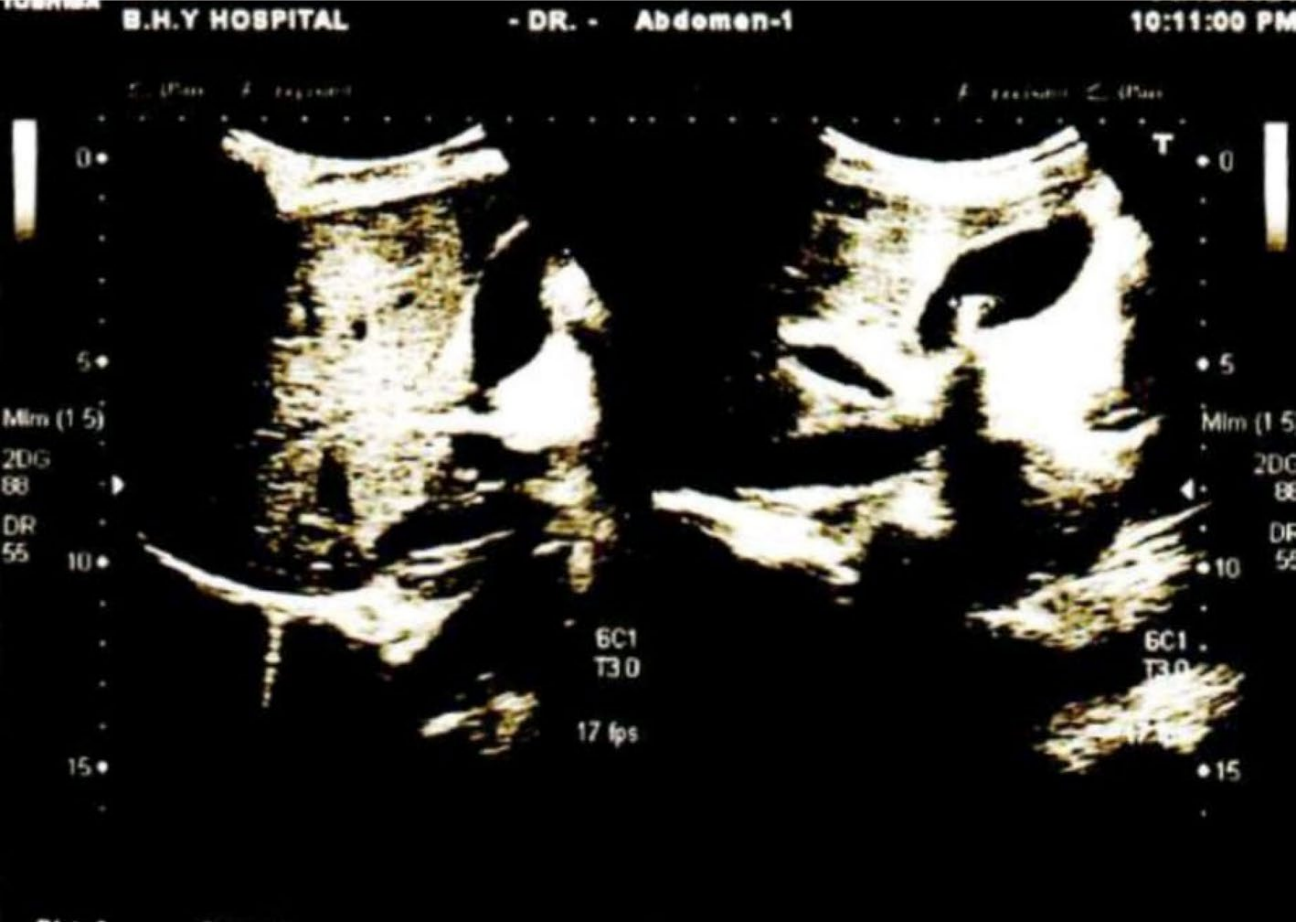
Abdominal ultrasonography showing the gallbladder in longitudinal view with multiple echogenic intraluminal calculi producing posterior acoustic shadowing. The adjacent liver appears normal in echotexture, with no sonographic evidence of biliary ductal dilatation.

### 2.3 Therapeutic Intervention

Given the high unconjugated bilirubin levels and clinical suspicion of CNS‐II, a therapeutic trial of oral phenobarbital was initiated. Phenobarbital was started empirically at a total daily dose of 180 mg, administered in two divided doses, after detailed counseling regarding potential risks and benefits. Liver function tests and bilirubin levels were monitored weekly following initiation of therapy.

Within 1 week, the patient's unconjugated bilirubin decreased by approximately 50%, indicating a significant response to phenobarbital (Figure 2). This phenobarbital responsiveness, in combination with the biochemical and clinical profile, supported the diagnosis of CNS‐II.

### 2.4 Follow‐Up and Outcomes

The patient was followed up after 4 weeks of therapy. His indirect bilirubin level decreased from 22.86 to 5.59 mg/dL and remained stable at this level thereafter, representing an overall reduction of nearly 70% from baseline (Figure 2). Clinically, he reported improvement in jaundice and overall well‐being (Figure 1b).

Based on the clinical course, laboratory trend, and robust response to phenobarbital, the diagnosis of CNS‐II was confirmed. After appropriate counseling regarding the benign but lifelong nature of the condition, avoidance of precipitating factors (such as fasting and hepatotoxic drugs), and the risk of pigment gallstone complications, the patient was referred to the surgical department for further evaluation and management of his cholelithiasis. The patient subsequently underwent an elective laparoscopic cholecystectomy, which revealed multiple pigment stones without biliary ductal dilatation. His postoperative course was uneventful. At 6‐month follow‐up, he remained asymptomatic from a biliary standpoint and continued low‐dose phenobarbital (60 mg once daily), with stable unconjugated bilirubin around 5–6 mg/dL and normal liver enzymes.

## 3 Discussion

Although the biochemical profile and phenobarbital responsiveness of our patient are typical for CNS‐II, this case is notable for three reasons: presentation in late adolescence after years of undiagnosed jaundice, the occurrence of symptomatic cholelithiasis at a young age, and confirmation of CNS‐II using a structured phenobarbital trial in the absence of genetic testing. These features underscore practical diagnostic and management challenges in resource‐limited settings.

Our patient's diagnostic workup demonstrated isolated, marked unconjugated hyperbilirubinemia with normal liver enzymes, normal synthetic function, and a negative hemolysis workup, fulfilling the classic biochemical pattern of CNS‐II (Table 1) [10]. In many reports, CNS‐II has been diagnosed on a clinical and biochemical basis, with phenobarbital responsiveness used as a functional test of residual UGT1A1 activity when genetic testing is unavailable [4, 11, 12]. Moreover, phenobarbital induction therapy can be used to clinically differentiate CNS‐I, CNS‐II, and Gilbert syndrome. In CNS‐II, phenobarbital induces residual UGT1A1 activity and typically produces a ≥ 25%–50% reduction in serum unconjugated bilirubin, whereas patients with CNS‐I show little or no change [13]. By contrast, patients with Gilbert syndrome have much lower baseline bilirubin levels, and phenobarbital commonly normalizes or near‐normalizes their bilirubin levels [14]. In practice, a supervised trial of phenobarbital for 1–2 weeks at standard doses, with careful monitoring of bilirubin trends and adverse effects, provides both diagnostic confirmation and therapeutic benefit in CNS‐II. In our patient, unconjugated bilirubin fell by nearly 70%, with clear improvement in scleral icterus, which provides strong functional evidence of CNS‐II, closely mirroring the response profile reported in other CNS‐II cases diagnosed without immediate access to genetic testing [4, 15]. The presence of gallstones in this patient is biologically and clinically relevant. Chronic unconjugated hyperbilirubinemia promotes calcium–bilirubinate precipitation and black pigment gallstone formation, making gallstone disease an under‐recognized complication of CNS‐II and other unconjugated hyperbilirubinemias [16]. In our case, ultrasound‐confirmed cholelithiasis explained his biliary symptoms, and elective cholecystectomy (with ongoing low‐dose phenobarbital) provided effective combined management.

This case has broader implications for clinicians practicing in diverse healthcare environments. In resource‐limited health systems, where UGT1A1 sequencing is not routinely available, a stepwise, clinically driven approach, documenting isolated unconjugated hyperbilirubinemia, excluding hemolysis and liver disease, considering family history and consanguinity, and performing a supervised phenobarbital trial, can prevent diagnostic delay and unnecessary, repeated investigations [4]. At the same time, in settings where genetic testing is readily available, knowing that typical lab findings, along with a clear drop in bilirubin after phenobarbital, strongly point to CNS‐II can help clinicians order genetic tests more selectively, reserving it for equivocal cases or family counseling; thus, reducing costs [6].

The main limitation of this report is the absence of a confirmatory *UGT1A1* genetic analysis. However, this limitation reflects a common reality in many low‐ and middle‐income regions, where the cost and availability of molecular testing remain significant barriers. Despite this constraint, our case adds value by illustrating a practical, reproducible diagnostic pathway for CNS‐II, highlighting the risk of early pigment gallstone disease, and emphasizing that a structured phenobarbital trial remains a powerful diagnostic and therapeutic tool where genomic confirmation is not readily available.

## 4 Conclusion

This case illustrates a rare presentation of CNS‐II in a young adult with lifelong unconjugated jaundice complicated by pigment gallstones. It highlights the need to consider CNS‐II in persistent unconjugated hyperbilirubinemia and supports the use of a supervised phenobarbital trial as a practical diagnostic and therapeutic tool, particularly where molecular testing is limited and to promote judicious use of genotyping when it is available. Early recognition, appropriate counseling, and long‐term follow‐up can prevent unnecessary investigations, enable timely management of biliary complications, and improve patients' quality of life.

## Author Contributions

**Danish Kumar Goswami:** conceptualization, investigation, writing – original draft. **Barkha Goswami:** methodology, writing – original draft. **Samiullah Shaikh:** conceptualization, writing – review and editing. **Abdul Ghani Rahimoon:** validation. **Rakesh Soni:** data curation. **Kamil Ahmad Kamil:** investigation.

## Funding

The authors have nothing to report.

## Ethics Statement

The authors have nothing to report.

## Consent

We explained everything to the patient in his native language, Sindhi, and obtained written informed consent for the publication of this case as per Wiley guidelines.

## Conflicts of Interest

The authors declare no conflicts of interest.

## Data Availability

Data sharing is not applicable to this article as no datasets were generated or analyzed during the current study.

## References

[1] J. Bhandari , P. K. Thada , M. Shah , and D. Yadav , Crigler‐Najjar Syndrome. StatPearls [Internet] (StatPearls Publishing, 2025), http://www.ncbi.nlm.nih.gov/books/NBK562171/. 32965842

[2] P. L. Jansen , “Diagnosis and Management of Crigler‐Najjar Syndrome,” European Journal of Pediatrics 158, no. Suppl 2 (1999): S89–S94, 10.1007/pl00014330. 10603107

[3] S. R. Fernandes , C. M. Moura , B. Rodrigues , L. A. Correia , H. Cortez‐Pinto , and J. Velosa , “Acute Cholangitis in an Old Patient With Crigler‐Najjar Syndrome Type II ‐ a Case Report,” BMC Gastroenterology 16 (2016): 33, 10.1186/s12876-016-0449-9. 26968162 PMC4788912

[4] A. Liaqat , A. Shahid , H. Attiq , A. Ameer , and M. Imran , “Crigler‐Najjar Syndrome Type II Diagnosed in a Patient With Jaundice Since Birth,” Journal of the College of Physicians and Surgeons‐Pakistan JCPSP 28 (2018): 806–808. 30266131

[5] M. Ciotti , S. L. Werlin , and I. S. Owens , “Delayed Response to Phenobarbital Treatment of a Crigler‐Najjar Type II Patient With Partially Inactivating Missense Mutations in the Bilirubin UDP‐Glucuronosyltransferase Gene,” Journal of Pediatric Gastroenterology and Nutrition 28 (1999): 210–213, 10.1097/00005176-199902000-00024. 9932859

[6] M. A. Rahman , M. S. U. Islam , and N. Tasnim , “Crigler Najjar Syndrome Type 2: A Case of Unexplained Jaundice in an Adult,” Faridpur Medical College Journal 15 (2020): 43–45, 10.3329/fmcj.v15i1.49011.

[7] CARE Case Report Guidelines [Internet] , “CARE Case Rep. Guidel,” https://www.care‐statement.org.

[8] K.‐H. Wagner , R. G. Shiels , C. A. Lang , N. Seyed Khoei , and A. C. Bulmer , “Diagnostic Criteria and Contributors to Gilbert's Syndrome,” Critical Reviews in Clinical Laboratory Sciences 55 (2018): 129–139, 10.1080/10408363.2018.1428526. 29390925

[9] L. M. Grant , T. W. Faust , V. Thoguluva Chandrasekar , and S. John , Gilbert Syndrome. StatPearls [Internet] (StatPearls Publishing, 2025), http://www.ncbi.nlm.nih.gov/books/NBK470200/. 29262099

[10] P. Kumar , G. Sasmal , S. Gupta , R. Saxena , and S. Kohli , “Crigler Najjar Syndrome Type 2 (CNS Type 2): An Unwonted Cause of Jaundice in Adults,” Journal of Clinical and Diagnostic Research : JCDR 11 (2017): OD05–OD06, 10.7860/JCDR/2017/28195.10221. PMC5583863 28892962

[11] “Diagnosis and management of Crigler‐Najjar syndrome ‐ PubMed [Internet],” https://pubmed.ncbi.nlm.nih.gov/10603107/.

[12] T. He , X. Geng , L. Zhu , X. Lin , and L. Wang , “Type II Crigler‐Najjar Syndrome: A Case Report and Literature Review,” Frontiers in Medicine 11 (2024): 1354514, 10.3389/fmed.2024.1354514. 38784231 PMC11112071

[13] A. Kadakol , S. S. Ghosh , B. S. Sappal , G. Sharma , J. R. Chowdhury , and N. R. Chowdhury , “Genetic Lesions of Bilirubin Uridine‐Diphosphoglucuronate Glucuronosyltransferase (UGT1A1) Causing Crigler‐Najjar and Gilbert Syndromes: Correlation of Genotype to Phenotype,” Human Mutation 16 (2000): 297–306, 10.1002/1098-1004(200010)16:4<297::AID-HUMU2>3.0.CO;2-Z. 11013440

[14] R. Sinha , S. Dalal , and K. Sodhi , “Differentiating Gilbert Syndrome From Crigler Najjar Syndrome Type 2 by Phenobarbitone Test,” Journal of Nepal Paediatric Society 35 (2015): 82–84.

[15] P. Beigvand , N. Moradi , S. Ramezani , and F. Firuzpour , “Delayed Diagnosis of Crigler‐Najjar Disease: A Case Report of a 17‐Year‐Old Man with Progressive Jaundice,” Case Reports in Clinical Practice [Internet] 9, no. 3 (2024): 114–118, https://crcp.tums.ac.ir/index.php/crcp/article/view/989.

[16] L. Vítek and M. C. Carey , “New Pathophysiological Concepts Underlying Pathogenesis of Pigment Gallstones,” Clinics and Research in Hepatology and Gastroenterology 36 (2012): 122–129, 10.1016/j.clinre.2011.08.010. 21978438 PMC3311771

